# Hypoxia and HIF activation as a possible link between sepsis and thrombosis

**DOI:** 10.1186/s12959-019-0205-9

**Published:** 2019-08-14

**Authors:** Colin E. Evans

**Affiliations:** 1 Program for Lung and Vascular Biology, Stanley Manne Children’s Research Institute, Ann & Robert H. Lurie Children’s Hospital of Chicago, Chicago, IL USA; 2 Department of Pediatrics, Division of Critical Care, Northwestern University Feinberg School of Medicine, Chicago, IL USA

**Keywords:** Endothelium, Hypoxia, Hypoxia-inducible factors, Integrins, Thrombosis

## Abstract

Risk factors for thrombosis include hypoxia and sepsis, but the mechanisms that control sepsis-induced thrombus formation are incompletely understood. A recent article published in Thrombosis Journal: (i) reviews the role of endothelial cells in the pathogenesis of sepsis-associated microthrombosis; (ii) describes a novel ‘two-path unifying theory’ of hemostatic discorders; and (iii) refers to hypoxia as a consequence of microthrombus formation in sepsis patients. The current article adds to this review by describing how sepsis and thrombus formation could be linked through hypoxia and activation of hypoxia-inducible transcription factors (HIFs). In other words, hypoxia and HIF activation may be a cause as well as a consequence of thrombosis in sepsis patients. While microthrombosis reduces microvascular blood flow causing local hypoxia and tissue ischemia, sepsis-induced increases in HIF1 activation could conversely increase the expression of coagulant factors and integrins that promote thrombus formation, and stimulate the formation of pro-thrombotic neutrophil extracellular traps. A better understanding of the role of cell-specific HIFs in thrombus formation could lead to the development of novel prophylactic therapies for individuals at risk of thrombosis.

Dear Editor,

Thrombosis is a common condition with potentially debilitating and fatal consequences, but the mechanisms that control thrombus formation are incompletely understood. Risk factors for thrombosis include trauma, pregnancy, high altitude, and sepsis [1]. Deep vein thrombosis occurs in regions of low blood flow, potentially leading to pulmonary embolism and post-thrombotic syndrome. The incidence of venous thrombosis is approximately 1 in 500 per year in the general population [2]. Arterial thrombi form under conditions of higher turbulent flow and thromboembolism can lead to fatal myocardial infarction or stroke. The major treatment for thrombosis is anticoagulation, which prevents thrombus extension, but increases the risk of bleeding [3]. Other treatments include thrombolysis and thrombectomy, but these are contraindicated in many patients, and carry increased risks of excessive bleeding and re-thrombosis [3]. Furthermore, clinical trials of anti-coagulants in sepsis patients have failed [4, 5]. A better understanding of the mechanisms that control thrombus formation could lead to the development of improved prophylactic therapies for individuals at risk for thrombosis, including but not limited to sepsis patients.

Sepsis-induced organ injury is commonly associated with the formation of small vessel microthrombi and an article in *Thrombosis Journal* has carefully reviewed the molecular mechanisms that control sepsis-induced microthrombosis with a focus on the endothelial cell response [5]. This recently-published review refers to hypoxia as a consequence of microthrombosis in sepsis [5], but it is also possible that hypoxia triggers microthrombus formation in sepsis patients [6, 7]. The review by Chang also refers to another article by the same author that describes the pathogenesis of disseminated intravascular microthrombosis and introduces a ‘two-path unifying theory’ of haemostatic disorders [8]. According to this theory, “normal” hemostasis is triggered by simultaneous but independent activation of tissue factor (TF) and “unusually large von Willebrand factor multimers”, while sepsis-associated endotheliopathy is triggered by activation of the unusually large von Willebrand factor multimers alone [5, 8]. In the more recent review article [5], Chang states that “DIC [disseminated intravascular coagulation] has been inappropriately conceptualized as a fibrin clot disease produced via activated TF/FVIIa-initiated cascade/cell-based coagulation” and that “consumption coagulopathy in acute promyelocytic leukaemia that occurs due to pathologic activation of aberrant TF path caused by TF released from leukemic promyelocytes should be called true DIC [8]”. The author also interestingly writes that “True DIC in acute promyelocytic leukaemia is made of disseminated fibrin clots that occur without vascular injury” [5]. Regardless of the terminology used for microthrombus formation or the setting in which thrombogenesis occurs, thrombus formation is a complex process that involves endothelial activation, integrin-mediated platelet-platelet and platelet-neutrophil aggregation, and formation of cross-linked fibrin [9]. These cellular processes are promoted by the adhesive functions of integrins, which are regulated at the levels of integrin expression, integrin activation through “inside-out” signalling, and post-ligand binding events through “outside-in” signalling [10].

The incidence of thrombosis is increased under conditions of hypoxia compared with normoxia [11, 12], and the more recent review by Chang refers to hypoxia as a characteristic of organ dysfunction in sepsis [5]. The vascular remodelling response to hypoxia is controlled primarily by hypoxia-inducible factors (HIF1 and HIF2) in nucleated cells [13]. Under hypoxia or following inflammatory challenges including the onset of sepsis, HIF1α and HIF2α (the hypoxia-dependent sub-units of HIF1 and HIF2, respectively) accumulate in the nucleus and form the active HIF1 or HIF2 complex, which bind to respective target genes and causes transcriptional upregulation. Endothelial HIF1 and HIF2 targets are distinct but overlapping and include factors that control coagulation, such as pro-thrombotic tissue factor (TF) and plasminogen activator inhibitor (PAI) 1 [13, 14]. Newly-formed large vein thrombus in murine inferior vena cava is severely hypoxic compared with venous blood [15] and the HIF1α and HIF2α isoforms are expressed in distinct spatial and temporal patterns within the newly-formed and resolving large vein thrombus as well as in the surrounding vein wall [15–17]. Furthermore, increases in the pulmonary levels of HIF1α and HIF2α expression occur in association with increases in pulmonary microthrombosis in mice [18]. These observations together suggest that increases in thrombus formation could be controlled by cell-specific HIFs, but despite evidence that systemic and endothelial hypoxia stimulate thrombosis [19, 20], the roles of endothelial cell-specific HIF1 and HIF2 in thrombus formation are unknown. Future studies should aim to assess the relative contributions of HIFs in different cell types to thrombus formation using genetically-altered mouse models and models of thrombosis [21–24].

Hypoxia increases the expression and function of endothelial integrin receptors, but the role of endothelial HIFs in the regulation of integrin function and expression is also unclear. It could be hypothesised that hypoxia or endothelial cell activation following sepsis challenge lead to HIF-dependent increases in the expression or function of hypoxia-inducible integrins, thereby stimulating thrombus formation. For example, hypoxia increases endothelial adhesion to fibrinogen via integrin αVβ3 and endothelial cell-cell interactions via αVβ3 and αVβ5 [25, 26]. The expression of cytoskeletal, focal adhesion, and cytosolic proteins that regulate integrin activation may also be affected by hypoxia in a HIF-dependent manner; these integrin-activating proteins include talin [27, 28], kindlin1–3 [29, 30], and Rap1 [31, 32]. Although it has been shown that endothelial expression of adhesive integrins can be increased by hypoxia [25, 26], experimental studies in future could aim to determine whether hypoxia and endothelial inflammation lead to increases in endothelial HIF1α and HIF2α, which in turn increase the expression or function of hypoxia-inducible integrins, and thereby promote thrombus formation. Such studies could aim to determine whether genes encoding hypoxia-regulated integrins contain the hypoxia-responsive element required for transcriptional upregulation by HIF binding. These studies could reveal a novel signalling pathway that controls thrombus formation and could ultimately lead to investigations of the effect of targeting such a pathway in humans. Although the positive associations between endothelial dysfunction, hypoxia, and thrombus formation in the septic microvasculature are well established and well described in the recent review by Chang [5], it is possible that hypoxia is both a consequence and cause of microthrombosis during sepsis and that a positive feedback loop exists between thrombus formation and inflammation in sepsis patients (Fig. 1). This possibility is consistent with the notion that pro-thrombotic pathways can also be pro-inflammatory and vice versa, but less consistent with the notion that inflammation does not alter activation of coagulation system or cause microthrombogenesis [5]. The proposal that thrombus formation could stimulate inflammation through hypoxia and HIF activation is also consistent with the observation that organ dysfunction in sepsis may be reversible [5], given that thrombus resolution is stimulated by hypoxia and activation of HIF1 and followed by temporal reductions in the severity of hypoxia and in the expression levels of HIF1α [15, 16, 33, 34]. Future studies should aim to improve understanding of the impact of varying severities of thrombosis on sepsis-induced inflammatory injury and of the mechanisms that control sepsis-induced thrombus formation. Such studies could again use genetically-altered mouse models in combination with experimental models of sepsis and thrombosis, for example in the pulmonary microvasculature [18], which is a common site of sepsis-induced organ dysfunction.

**Fig. 1:**
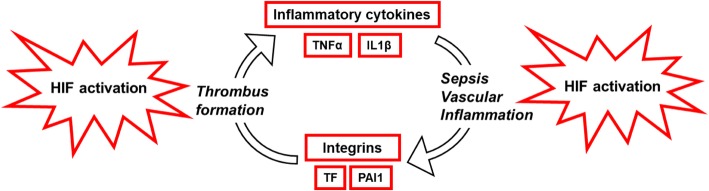
HIF activation as the link between thrombus formation and inflammatory vascular injury

Thrombus formation leads to hypoxia and HIF activation, resulting in the release of inflammatory factors that promote vascular inflammation, while sepsis challenge triggers HIF activation and increases the expression of integrins and coagulant factors.

Another mechanism through which hypoxia and HIF activation could promote thrombosis during sepsis is via increases in the formation of neutrophil extracellular traps (NETs) [35]. NETs are DNA fibres comprising of histones and antimicrobial proteins that are formed within the septic vasculature and promote thrombus formation [36, 37]. In his recent review, Chang postulates that “NETosis is not active hemostatic processes, but is a passive one associated with secondary event trapping blood cells and molecules such as DNAs and histones in the process of thrombogenesis” [5]. However, inflammatory and hypoxic conditions trigger HIF1 and NET activation [37, 38], and it has also been suggested that NETs themselves increase endothelial activation [39, 40], thus creating another putative thrombo-inflammatory positive feedback loop. Furthermore, it has been shown that NET formation is regulated by post-transcriptional control of HIF1α expression following sepsis challenge and that pharmacological or genetic knockdown of HIF1α inhibits NET deployment [35]. So, while our understanding of sepsis-induced thrombus formation has improved substantially in recent years, future studies investigating the signalling pathways that control thrombus formation could eventually identify novel therapeutic targets and aid in the development of new therapies against thrombosis.

## Data Availability

N/A.

## Abbreviations

HIF: Hypoxia-inducible factor
NETs: Neutrophil extracellular traps

## References

[1] Mackman N (2012). Mouse models, risk factors, and treatments of venous thrombosis. Arterioscler Thromb Vasc Biol.

[2] Fowkes FJ, Price JF, Fowkes FG (2003). Incidence of diagnosed deep vein thrombosis in the general population: systematic review. Eur J Vasc Endovasc Surg.

[3] Rectenwald JE, Wakefield TW (2005). The treatment of deep venous thrombosis, including the newer agents. Dis Mon.

[4] Evans CE, Zhao YY (2017). Impact of thrombosis on pulmonary endothelial injury and repair following sepsis. Am J Physiol Lung Cell Mol Physiol.

[5] Chang JC (2019). Sepsis and septic shock: endothelial molecular pathogenesis associated with vascular microthrombotic disease. Thromb J.

[6] Gupta N, Zhao YY, Evans CE. The stimulation of thrombosis by hypoxia. Thromb Res. 2019. 10.1016/j.thromres.2019.07.013 31376606

[7] Evans Colin E., Spier Addie B., Zhao You-Yang (2018). Sepsis-induced thrombus formation and cell-specific HIFs. Thrombosis Research.

[8] Chang JC (2018). Hemostasis based on a novel 'two-path unifying theory' and classification of hemostatic disorders. Blood Coagul Fibrinolysis.

[9] Saha P, Humphries J, Modarai B, Mattock K, Waltham M, Evans CE, Ahmad A, Patel AS, Premaratne S, Lyons OT, Smith A (2011). Leukocytes and the natural history of deep vein thrombosis: current concepts and future directions. Arterioscler Thromb Vasc Biol.

[10] Shattil SJ, Kim C, Ginsberg MH (2010). The final steps of integrin activation: the end game. Nat Rev Mol Cell Biol.

[11] Hamer JD, Malone PC, Silver IA (1981). The PO2 in venous valve pockets: its possible bearing on thrombogenesis. Br J Surg.

[12] Yan SF, Mackman N, Kisiel W, Stern DM, Pinsky DJ (1999). Hypoxia/hypoxemia-induced activation of the procoagulant pathways and the pathogenesis of ischemia-associated thrombosis. Arterioscler Thromb Vasc Biol.

[13] Semenza GL (2010). Vascular responses to hypoxia and ischemia. Arterioscler Thromb Vasc Biol.

[14] Liao H, Hyman MC, Lawrence DA, Pinsky DJ (2007). Molecular regulation of the PAI-1 gene by hypoxia: contributions of Egr-1, HIF-1alpha, and C/EBPalpha. FASEB J.

[15] Evans CE, Humphries J, Mattock K, Waltham M, Wadoodi A, Saha P, Modarai B, Maxwell PH, Smith A (2010). Hypoxia and upregulation of hypoxia-inducible factor 1{alpha} stimulate venous thrombus recanalization. Arterioscler Thromb Vasc Biol.

[16] Evans CE, Humphries J, Waltham M, Saha P, Mattock K, Patel A, Ahmad A, Wadoodi A, Modarai B, Burnand K, Smith A (2011). Upregulation of hypoxia-inducible factor 1 alpha in local vein wall is associated with enhanced venous thrombus resolution. Thromb Res.

[17] Evans CE, Wadoodi A, Humphries J, Lu X, Grover SP, Saha P, Smith A (2014). Local accumulation of hypoxia-inducible factor 2 alpha during venous thrombus resolution. Thromb Res.

[18] Evans CE, Palazon A, Sim J, Tyrakis PA, Prodger A, Lu X, Chan S, Bendahl PO, Belting M, Von Euler L (2017). Modelling pulmonary microthrombosis coupled to metastasis: distinct effects of thrombogenesis on tumorigenesis. Biol Open.

[19] Brill A, Suidan GL, Wagner DD (2013). Hypoxia, such as encountered at high altitude, promotes deep vein thrombosis in mice.

[20] Ogawa S, Gerlach H, Esposito C, Pasagian-Macaulay A, Brett J, Stern D (1990). Hypoxia modulates the barrier and coagulant function of cultured bovine endothelium**.** Increased monolayer permeability and induction of procoagulant properties. J Clin Invest.

[21] Evans CE, Bendahl PO, Belting M, Branco C, Johnson RS (2016). Diverse roles of cell-specific hypoxia-inducible factor 1 in cancer-associated hypercoagulation. Blood.

[22] Evans CE, Humphries J, Saha P, Smith A (2012). Opinions on mouse models of thrombosis. Thromb Res.

[23] Diaz JA, Saha P, Cooley B, Palmer OR, Grover SP, Mackman N, Wakefield TW, Henke PK, Smith A, Lal BK (2019). Choosing a mouse model of venous thrombosis: a consensus assessment of utility and application. J Thromb Haemost.

[24] Evans CE (2016). Inducing femoral vein thrombosis under unrestricted flow: comments on an alternative electrolytic mouse model. Thromb Res.

[25] Walton HL, Corjay MH, Mohamed SN, Mousa SA, Santomenna LD, Reilly TM (2000). Hypoxia induces differential expression of the integrin receptors alpha (vbeta3) and alpha (vbeta5) in cultured human endothelial cells. J Cell Biochem.

[26] Niu JX, Zhang WJ, Ye LY, Wu LQ, Zhu GJ, Yang ZH, Grau GE, Lou JN (2007). The role of adhesion molecules, alpha v beta 3, alpha v beta 5 and their ligands in the tumor cell and endothelial cell adhesion. Eur J Cancer Prev.

[27] Liu X, Schnellmann RG (2003). Calpain mediates progressive plasma membrane permeability and proteolysis of cytoskeleton-associated paxillin, Talin, and vinculin during renal cell death. J Pharmacol Exp Ther.

[28] Wegener KL, Partridge AW, Han J, Pickford AR, Liddington RC, Ginsberg MH, Campbell ID (2007). Structural basis of integrin activation by Talin. Cell.

[29] Karakose E, Schiller HB, Fassler R (2010). The kindlins at a glance. J Cell Sci.

[30] Shen Z, Ye Y, Kauttu T, Seppanen H, Vainionpaa S, Wang S, Mustonen H, Puolakkainen P (2013). The novel focal adhesion gene kindlin-2 promotes the invasion of gastric cancer cells mediated by tumor-associated macrophages. Oncol Rep.

[31] Lee JW, Ryu YK, Ji YH, Kang JH, Moon EY (2015). Hypoxia/reoxygenation-experienced cancer cell migration and metastasis are regulated by Rap1- and Rac1-GTPase activation via the expression of thymosin beta-4. Oncotarget.

[32] Aslam M, Schluter KD, Rohrbach S, Rafiq A, Nazli S, Piper HM, Noll T, Schulz R, Gunduz D (2013). Hypoxia-reoxygenation-induced endothelial barrier failure: role of RhoA, Rac1 and myosin light chain kinase. J Physiol.

[33] Evans CE, Humphries J, Mattock K, Saha P, Smith A (2012). HIF1 signalling regulates venous thrombus resolution. Thromb Res.

[34] Evans CE, Humphries J, Waltham M, Saha P, Mattock K, Patel A, Ahmad A, Wadoodi A, Modarai B, Burnand K, Smith A (2012). Adenoviral delivery of constitutively active HIF1alpha into venous thrombus. Thromb Res.

[35] McInturff AM, Cody MJ, Elliott EA, Glenn JW, Rowley JW, Rondina MT, Yost CC (2012). Mammalian target of rapamycin regulates neutrophil extracellular trap formation via induction of hypoxia-inducible factor 1 alpha. Blood.

[36] Fuchs TA, Brill A, Duerschmied D, Schatzberg D, Monestier M, Myers DD, Wrobleski SK, Wakefield TW, Hartwig JH, Wagner DD (2010). Extracellular DNA traps promote thrombosis. Proc Natl Acad Sci U S A.

[37] Clark SR, Ma AC, Tavener SA, McDonald B, Goodarzi Z, Kelly MM, Patel KD, Chakrabarti S, McAvoy E, Sinclair GD (2007). Platelet TLR4 activates neutrophil extracellular traps to ensnare bacteria in septic blood. Nat Med.

[38] De Meyer SF, Suidan GL, Fuchs TA, Monestier M, Wagner DD (2012). Extracellular chromatin is an important mediator of ischemic stroke in mice. Arterioscler Thromb Vasc Biol.

[39] Brill A, Fuchs TA, Savchenko AS, Thomas GM, Martinod K, De Meyer SF, Bhandari AA, Wagner DD (2012). Neutrophil extracellular traps promote deep vein thrombosis in mice. Journal of thrombosis and haemostasis : JTH.

[40] Martinod K, Wagner DD (2014). Thrombosis: tangled up in NETs. Blood.

